# Deep learning-based automated detection of Micro-cracks in monolithic zirconia crowns using Micro-CT imaging: An *in vitro* study

**DOI:** 10.6026/973206300222694

**Published:** 2026-04-30

**Authors:** Gurdeep Kaur Chauhan, Ravi Ranjan Sinha, Rohit Mehta, Upasana Chhabra, Shailesh D Gawande, Pooja Arora

**Affiliations:** 1 Department of Prosthodontics and Crown & Bridge, the Oxford Dental College, Bangalore, India; 2 Department of Prosthodontics Crown and Bridge, Geetanjali Dental & Research institute, Udaipur, Rajasthan, India; 3 Department of Dentistry, Government Medical College and Hospital, Ratnagiri, India; 4 Department of Prosthodontics, Taif University, Saudi Arabia

**Keywords:** Deep learning (DL), micro-crack detection, monolithic zirconia, micro CT, convolutional neural network (CNN)

## Abstract

Early detection of Micro-cracks in monolithic zirconia crowns remains a challenge because conventional inspection methods cannot identify subsurface defects that may lead to clinical failure. Therefore, it is of interest to develop and evaluate a deep learning convolutional neural network model for detecting and classifying Micro-cracks in zirconia crowns using high-resolution micro-computed tomography imaging. Hence, sixty zirconia crowns were fabricated and divided into control and experimentally stressed groups, generating 1,440 labeled micro-CT cross-sectional images that were used to train (80%) and test (20%) a ResNet-50 model. The model achieved an overall accuracy of 94.7%, sensitivity of 93.2%, specificity of 96.1% and an AUC of 0.97 for micro-crack detection and classification. Deep learning combined with micro-CT imaging provides a highly accurate and automated approach for identifying Micro-cracks in zirconia crowns, with potential to enhance quality assurance in dental manufacturing workflows.

## Background:

In the recent past, due to the combination of its outstanding mechanical characteristics, such as flexural strength of over 1000 Mpa, high fracture toughness and excellent wear resistance and to increasing degrees of translucency, offering potentially satisfactory esthetic results in the absence of any porcelain veneering layers, monolithic zirconia has become one of the most popular materials in modern fixed prosthodontics [1]. The problematic complication of chipping and delamination that afflicted bilayered zirconia restorations in the past is removed by the use of the veneering ceramic making monolithic zirconia an especially appealing material in the anterior full-coverage crowns and fixed dental prostheses [2]. The results of clinical studies have indicated good survival rates greater than 95 at intervals of five years and this has supported the material on evidence-based prosthodontic practice [3]. In spite of these positive features, monolithic zirconia is not free of the structural degradation. Micro-cracks are microscale discontinuities in the microstructure of ceramics (i.e., 10 to 50,000:00): Micro-cracks may form at various points throughout the lifecycle of the restoration, such as CAD/CAM milling, sintering, surface adjustment, sandblasting and long-term intraoral service under cyclic masticatory loading [4]. These Micro-cracks are points of stress concentration that may further grow when subjected to further mechanical and thermal cycling by the process of subcritical crack growth which may culminate into the catastrophic fracture of the material at loads significantly lower than the nominal flexural strength of the material [5]. Clinical significance of Micro-cracks is further enhanced due to the observation of low temperature degradation whereby moisture-aided conversion of tetragonal to monoclinic phase transformation at the end of the cracks increases the speed of the crack propagation in oral environment [6]. Micro-cracks on dental ceramic restorations are difficult to detect and a big technical challenge. Quality evaluation techniques currently used in dental laboratory and practice environments, e.g., eye inspection, transillumination, as well as the haptic sense with dental explorers are restricted to the detection of the macroscopic surface defects and are inherently unable to detect Micro-cracks that may be present under the surface in the bulk of the restoration [7]. More advanced methods such as scanning electron microscopy, optical coherence tomography and acoustic emission monitoring are also examined in detecting cracks in dental ceramics but because all are destructive in nature, prohibitively time-consuming or lack the capability of providing detailed three-dimensional characterization of the crack network [8]. Micro-computed tomography has now proven to be an effective non-destructive imaging modality that can produce very high-resolution three-dimensional volumetric images of internal material structure at micrometer-level resolution [9]. Micro-CT has been effectively utilized in the dental materials studies to assess internal porosity, marginal adaptation, cement layer thickness and interfacial defects in ceramic restorations [10]. Micro-crack detection by micro-CT in zirconia is theoretically possible, since the high-density ceramic matrix and the empty space of the crack offer enough contrast, but the very high volumes of volumetric data produced during high- resolution scanning can pose a challenge to interpretation by hand since the closed-circuit system can be provided with thousands of cross- sectional images at a time [11]. The appearance of deep learning a branch of artificial intelligence relying on multi-layered artificial neural networks has transformed automated image analysis in a variety of biomedical fields [12]. Convolutional neural networks, specifically, have shown impressive ability to work at medical image classification, segmentation and object detection tasks and show performance levels that rival or surpass those of expert human observers in tasks such as histopathological diagnosis or radiographic interpretation tasks [13]. Deep learning has also been applied in the field of dentistry to perform automated tooth caries, periapical lesions and periodontal bone loss and implant classification, noticed in panoramic and periapical radiographs [14]. Nevertheless, investigating the use of deep learning in the quality evaluation of dental materials, namely, the automatic identification of structural flaws in ceramic restorations is not well studied [15]. The combination of high-resolution micro-CT imaging with the deep learning-based image analysis offers the interesting chance to create automated, objective and reproducible methods of detecting Micro-cracks in dental ceramics. This system has the potential to revolutionize the workflows of quality control of digital dental manufacturing through facilitating the quick screening of restorations in terms of structural defects before the clinical provision, therefore, diminishing the number of premature mechanical failures and enhancing patient results [16]. Nevertheless, training systems of this kind have to be developed by creating training datasets that are well-annotated, by identifying and training the right neural network architectures and by performing extensive testing of diagnostic output with expert ground truth [17]. Therefore, it is of interest to develop and evaluate a deep learning-based model for automated detection and classification of Micro-cracks in monolithic zirconia crowns using micro-CT imaging.

## Materials and Methods:

## Study design overview:

This *in vitro* diagnostic accuracy study comprised four sequential phases: (1) specimen fabrication and controlled aging, (2) micro-CT image acquisition, (3) expert annotation and ground truth establishment and (4) deep learning model development and validation. The study was conducted at the digital dentistry research laboratory and the biomedical imaging center of the Faculty of Dentistry.

## Specimen fabrication:

Sixty monolithic zirconia crowns were fabricated for maxillary first premolar preparations. A standardized master die was designed digitally using CAD software (3Shape Dental System, 3Shape A/S, Copenhagen, Denmark) with the following preparation parameters: 1.5 mm occlusal reduction, 1.0 mm axial reduction, 6°C total convergence angle and 1.0 mm deep chamfer finish line. The digital die design was duplicated to produce sixty identical polyurethane dies (AlphaDie MF, Schutz Dental, Germany). Crown restorations were designed digitally using the same CAD software with standardized parameters: 1.5 mm minimum occlusal thickness 1.0 mm minimum axial wall thickness and 30 µm cement space. All crowns were milled from pre-sintered 3Y-TZP zirconia blanks (Katana Zirconia STML, Kuraray Noritake, Japan) using a five-axis milling machine (DWX-52D, Roland DG, Japan). After milling, all crowns underwent sintering according to the manufacturer's protocol (final sintering at 1500°C for 2 hours with controlled heating and cooling rates).

## Specimen grouping and aging protocol:

Following fabrication, all crowns underwent visual and microscopic inspection (stereomicroscope, 20 x magnifications) to exclude specimens with visible fabrication defects. Crowns were randomly allocated into three groups:

[1] Group A - Control (n = 20): No aging treatment; stored in dry conditions at room temperature

[2] Group B - Thermocycled (n = 20): Subjected to 10,000 thermal cycles between 5°C and 55°C with 30-second dwell time and 5-second transfer time in a thermocycling apparatus (THE-1100, SD Mechatronik, Germany)

[3] Group C - Thermo mechanically Aged (n = 20): Subjected to 10,000 thermal cycles (identical protocol as Group B) followed by 1,200,000 mechanical loading cycles at 200 N, 2 Hz frequency, in a chewing simulator (CS-4.8, SD Mechatronik) with the crown cemented on its respective die using resin cement (RelyX Ultimate, 3M, USA) and loaded through a stainless steel antagonist sphere (6 mm diameter) in artificial saliva at 37°C

## Micro-CT image acquisition:

All sixty crowns were scanned using a high-resolution micro-CT system (SkyScan 1272, Bruker, Belgium) with the following acquisition parameters:

[1] Source voltage: 100 kV

[2] Source current: 100 µA

[3] Voxel size: 9 µm isotropic

[4] Rotation step: 0.2°C

[5] Frame averaging: 4 frames

[6] Filter: 0.11 mm copper

[7] Scan duration: Approximately 3.5 hours per specimen

[8] Total rotation: 360°C

Raw projection images were reconstructed into cross-sectional slices using the manufacturer's reconstruction software (NRecon, Bruker) with standardized parameters including beam hardening correction (40%), ring artifact reduction (5) and smoothing (1). Each crown scan yielded approximately 240 cross-sectional grayscale images (16-bit TIFF format, 2016 x 2016 pixels), resulting in a total of 14,400 images across all sixty specimens.

## Expert annotation and ground truth establishment:

All 14,400 cross-sectional images were independently reviewed and classified by two expert examiners (one dental materials scientist with 12 years of experience in ceramic microstructure analysis and one biomedical imaging specialist with 8 years of micro-CT expertise). Each image was classified into one of three categories:

[1] Class 0 - No crack: No visible linear discontinuity or void suggestive of a Micro-crack

[2] Class 1 - Minor Micro-crack: Linear discontinuity measuring less than 100 µm in length, not extending through more than 25% of the wall thickness

[3] Class 2 - Major Micro-crack: Linear discontinuity measuring 100 µm or greater in length, or extending through more than 25% of the wall thickness

Images with disagreement between the two examiners were reviewed jointly and a consensus classification was established. Inter-examiner agreement was assessed using Cohen's kappa statistic. The consensus annotations served as the ground truth labels for deep learning model training and validation.

## Image pre-processing:

Prior to model training, all images underwent standardized pre-processing:

[1] Region of interest cropping to isolate the crown cross-section from the background

[2] Resizing to 224 x 224 pixels (input dimensions for ResNet-50)

[3] Intensity normalization to the 0-1 range

[4] Data augmentation applied to the training set: random horizontal and vertical flipping, rotation (±15°C), brightness adjustment (±10%) and Gaussian noise addition (σ = 0.01)

## Deep learning model architecture and training:

ResNet-50 convolutional neural network architecture was selected for the classification task. The model was initialized with weights pre-trained on the ImageNet dataset (transfer learning) and fine-tuned for the three-class Micro-crack classification task. The final fully connected layer was modified to output three class probabilities via a softmax activation function. The dataset was split into training (80%, n = 11,520 images) and validation (20%, n = 2,880 images) sets using stratified random sampling to maintain proportional class representation in both sets. The split was performed at the specimen level (not image level) to prevent data leakage between training and validation sets - all images from a given crown were allocated entirely too either the training or validation set.

## Training hyper-parameters:

[1] Optimizer: Adam (learning rate: 0.0001, β1 = 0.9, β2 = 0.999)

[2] Loss function: Categorical cross-entropy

[3] Batch size: 32

[4] Epochs: 100 with early stopping (patience = 10 epochs based on validation loss)

[5] Class weighting: Inversely proportional to class frequency to address class imbalance

[6] Regularization: Dropout (rate = 0.5) in fully connected layers; L2 weight decay (λ = 0.0001)

Training was performed on an NVIDIA RTX 3090 GPU (24 GB VRAM) using PyTorch framework (version 1.12). Training completed in 67 epochs before early stopping was triggered.

## Performance evaluation metrics:

Model performance on the validation set was assessed using the following metrics:

[1] Overall accuracy: Proportion of correctly classified images across all three classes

[2] Per-class sensitivity (recall): Proportion of true positives correctly identified within each class

[3] Per-class specificity: Proportion of true negatives correctly identified for each class

[4] Per-class precision (positive predictive value)

[5] F1 score: Harmonic mean of precision and recall for each class

[6] Area under the receiver operating characteristic curve (AUC): Calculated using one-versus-rest approach for multi-class classification

[7] Confusion matrix: Tabulation of predicted versus true classifications

Additionally, Grad-CAM (Gradient-weighted Class Activation Mapping) visualizations were generated for a subset of correctly and incorrectly classified images to provide visual interpretation of the features driving model predictions.

## Statistical analysis:

Statistical analysis was performed using Python (version 3.9) with SciPy (version 1.9) and scikit-learn (version 1.1) libraries. Descriptive statistics were computed for all performance metrics. The 95% confidence intervals for accuracy, sensitivity and specificity were calculated using the Wilson score method. McNemar's test was used to compare the model's classification performance against each individual expert examiner. Chi-square tests were used to compare crack prevalence among specimen groups. Statistical significance was set at p < 0.05.

## Results:

Inter-examiner agreement for image classification was substantial, with a Cohen's kappa value of 0.87, indicating high reproducibility between examiners. After consensus review, the distribution of ground truth image classifications across the three specimen groups is presented in Table 1 (see PDF). Class 0 images, representing no crack, were most frequently observed in Group A (92.3%), followed by Group B (79.0%) and Group C (52.0%). In contrast, Micro-crack prevalence increased progressively from Group A to Group C. Minor Micro-cracks were observed in 6.8% of Group A images, 15.5% of Group B images, and 26.6% of Group C images. Major Micro-cracks were least frequent in Group A (0.9%) and highest in Group C (21.4%). Overall, Group C demonstrated the highest combined Micro-crack prevalence, followed by Group B and Group A. The deep learning model classification performance on the validation set is shown in Table 2 (see PDF). The model achieved an overall accuracy of 94.7%. The highest sensitivity was observed for Class 0 images (97.3%), followed by Class 2 images (93.8%) and Class 1 images (86.7%). Specificity was highest for Class 2 (98.6%), followed by Class 1 (97.2%) and Class 0 (91.4%). Precision values were 96.2% for Class 0, 88.4% for Class 1, and 94.7% for Class 2. The weighted average sensitivity, specificity, precision, and F1 score were 93.2%, 96.1%, 93.6%, and 0.940, respectively. The AUC values were high across all classes, with the highest value recorded for Class 2 (0.987), followed by Class 0 (0.983) and Class 1 (0.954). The confusion matrix for the validation set is presented in Table 3 (see PDF). Of the 2,145 actual Class 0 images, 2,087 were correctly classified, while 52 were misclassified as Class 1 and 6 as Class 2. Among the 470 actual Class 1 images, 408 were correctly classified, whereas 48 were misclassified as Class 0 and 14 as Class 2. For Class 2 images, 249 of 265 were correctly classified, with 12 misclassified as Class 1 and 4 as Class 0. The most common misclassification occurred between adjacent categories, particularly between Class 0 and Class 1. Misclassification between non-adjacent classes was uncommon. McNamara's test comparing binary crack detection performance of the deep learning model with individual expert examiners showed no statistically significant difference, indicating comparable diagnostic performance. Gradient-weighted class activation mapping demonstrated that the model's attention was generally concentrated along Micro-crack regions identified by expert examiners. False-negative cases were commonly associated with peripheral or low-contrast Micro-cracks, whereas false-positive cases were mainly related to sintering pores or surface irregularities mimicking Micro-crack patterns.

## Discussion:

The current research is the initial systematic analysis of the application of deep learning in automated Micro-crack detection in monolithic zirconia crowns with micro-CT images. An overall classification accuracy of the developed ResNet-50 model was 94.7 with an AUC of 0.97 indicating that deep learning is able to correctly identify intact and Micro-cracked regions in high-resolution cross-sectional micro-CT images of dental ceramic restorations. The null hypothesis is conclusively rejected and the possibility of artificially establishing the quality of artificial intelligence-enhanced structural assessment of dental ceramics is confirmed. The recorded overall accuracy of 94.7% and the weighted F1 score of 0.94 are relatively favorable in comparison to the benchmarks of deep learning based on biomedical image classification tasks. Medical imaging in medical imaging, classification rates of 90-95 percent are usually viewed as clinically useful in screening cases [18]. The diagnostic accuracy of the given study is especially remarkable considering the complexity of the problem of classification of Micro-cracks on zirconia are observed as little linear discontinuities with minimal contrast between them and the solid ceramic and their distinction against the imaging artifacts, sintering porosity and noise demands advanced pattern recognition abilities [19]. The comparison of the performance of the three classification categories offers valuable information on the strengths and limitations of the model. Class 0 (no crack, 97.3% was the most sensitive, which is not surprising as intact ceramic is a relatively homogenous and distinct object that the network learns to identify quite easily. Class 2 (major Micro-crack, 0.987) had the largest per-class AUC, which suggested that bigger structural discontinuities generate imaging signatures distinctive enough to be well detected in an automated manner. The Class 1 (minor Micro-crack, 86.7) has a lower sensitivity which is due to the real diagnostic difficulty of subtle Micro-cracks that border on the resolution limits of the imaging system and has a low contrast difference with the surrounding matrix [20]. The analysis of the confusion matrix showed that the most common misclassification tendency was the confusion of neighbour classes and not non-neighbour classes, which implies that the model reflects the ordinality of the classification scheme and makes clinical sensible mistakes when they happen. The type of error with the most clinically relevant consequences is the misclassification of 48 minor Micro-crack images as being crack-free (10.2% of Class 1 images), because this type of error is false-negative diagnoses and may result in subtly damaged restorations passing through quality assessment. These false negatives were however found to be mainly linked to Micro-cracks at detection limit (about 10-30 mm in length) which might not be clinically significant structural compromise [21]. The data on the Micro-crack prevalence produced by expert annotation offer an excellent understanding of the structural consequences of the aging on monolithic zirconia. The fact that thermomechanical aging induced Micro-cracks in 48 percent of the cross-sectional images compared to 21 percent when subjected to thermal cycling alone and 7.7 percent in unaged controls confirm that cyclic mechanical loading is a stronger initiator of Micro-crack formation and propagation when compared to thermal cycling alone in monolithic zirconia [22]. This observation is in line with the principles of fatigue mechanics, in which repetitive mechanical loads produce successive crack nucleation at existing microstructural discontinuities including grain boundaries, pores and surface defects caused by machining [23]. The ResNet-50 architecture was chosen due to its proven efficiency in the medical image classification problems and its positive balance between the model complexity and the efficiency of its computations. The residual learning framework solves the issue of degradation that comes with very deep networks that have allowed effective extraction of features of the fine textural features that come with Micro-cracks [24]. ImageNet pre-training transfer learning gave beneficial initialization of the convolutional filters, rapid convergence and better generalization despite the relatively small size of the dataset by the standards of dental imaging [25]. The use of class-weighted loss function effectively overcomes the existing imbalance in number of classes in the dataset which caused the model to become biased towards the majority class at the cost of sensitivity of Micro-crack detection. Grad-CAM visualization analysis has proven the hypothesis that the model decision-making was informed by anatomically and structurally significant features and not by spurious correlations or artifacts in the image. The fact that model attention is localized to the real cracks gives the faith that the learned representations are rich and can be interpreted, which is a major concern when it comes to the translational acceptability of artificial intelligence tools in healthcare applications [26]. It is because the sintering pores were found to be a frequent source of false-positive classification that further training data containing explicit pore-annotations may enhance the discriminative capacity of the model. The effective application of this technology to dental manufacturing quality controls is extreme. Most recent quality evaluation of milled zirconia restorations is based on visual inspection with the aid of marginal fit assessment, which does not allow detecting internal structural flaws [27]. Combining micro-CT scanning with an analysis system based on deep learning may allow screening high-value restorations, including implant- supported crowns and extended fixed dental prostheses, automatically to internal manufacturing errors before clinical delivery. Although the scanning time currently required (around 3.5 hours per specimen) of the protocol is unfeasible in practice due to its high cost, continued development of photon-counting detector technology and iterative reconstruction algorithms is steadily lowering micro-CT acquisition times to the range of clinically feasible times [28]. Moreover, the constructed methodology can be used in other areas other than quality control. Automated Micro-crack quantification in dental materials science has the potential to make high-throughput assessment of aging regimes, surface treatment responses and optimization of material formulations and so on. Another application of the deep learning-based crack characterization in failure analysis is to retrospective micro-CT scan clinically failed restorations with deep learning to offer insight into failure modes and to guide material development [29]. There are a number of limitations that should be considered. Although *in vitro* study design offers control experimental conditions, it does not completely mimic the complexity of the oral environment of saliva exposure, pH changes and variable loading vectors. The elastic behavior of natural dentin and periodontal ligament are not recreated by the synthetic bone analog dies and this could affect the transmission of stress and crack patterns during loading [30]. The smallest voxel micro-CT size of 90 nm defines a theoretical minimum cut-off point in detecting cracks and Micro-cracks less than voxel dimensions that lead to the material degradation may go undetected. The model was established on one type of zirconia brand and morphology of its crown and its externalizability to other zirconia formulas, translucency levels, restoration geometries and micro-CTs needs to be explored further [31]. Also, a hold-out subset of the same population of the experiment was used to validate, external validation with independent datasets would enhance the belief in the clinical usefulness of the model. Future directions in research should incorporate the inclusion of more zirconia formulations and types of restoration, the exploration of three-dimensional volumetric segmentation methods exploring the spatial continuity of cracks between adjacent slices, the production of lightweight model architectures that can be implemented in common clinical computing systems and future correlations of micro-CT identified Micro-cracks with clinical fracture occurrence [32].

## Conclusion:

Deep learning using a ResNet-50 convolutional neural network demonstrated high accuracy in detecting and classifying Micro-cracks in monolithic zirconia crowns from micro-CT images, with performance comparable to expert evaluation. The model showed highest reliability for major defects, while thermomechanical aging significantly increased Micro-crack formation, highlighting fatigue as a key factor in structural degradation. Integration of micro-CT imaging with deep learning offers a promising, objective approach for detecting subsurface defects and enhancing quality control in dental restorative materials.
